# Weisheng-Tang Ameliorates Acute Ischemic Brain Damage in Mice by Maintaining Blood-Brain Barrier Integrity

**DOI:** 10.1155/2019/4379732

**Published:** 2019-12-03

**Authors:** Min Jae Kim, Ki Hyun Park, Ji Yun Lee, Ki-Tae Ha, Byung Tae Choi, Jin Ung Baek, Young Ju Yun, Seo-Yeon Lee, Hwa Kyoung Shin

**Affiliations:** ^1^Department of Korean Medical Science, School of Korean Medicine, Pusan National University, Yangsan, Gyeongnam 50612, Republic of Korea; ^2^Korean Medical Science Research Center for Healthy-Aging, Pusan National University, Yangsan, Gyeongnam 50612, Republic of Korea; ^3^Graduate Training Program of Korean Medicine for Healthy-Aging, Pusan National University, Yangsan, Gyeongnam 50612, Republic of Korea; ^4^Department of Korean Medicine, School of Korean Medicine, Pusan National University, Yangsan, Gyeongnam 50612, Republic of Korea

## Abstract

Stroke is one of the major causes of death and long-term disability worldwide; the associated breakdown of the blood-brain barrier (BBB) aggravates ischemic brain damage. Accordingly, many medicinal herbs and formulas have been used to treat stroke-related symptoms. In this study, we selected two Korean herbal medicine formulas, Weisheng-tang and Tongxuewan, through *Dongeuibogam* text-mining analysis, and evaluated their protective effect on BBB disruption and brain damage in stroke. Ischemic brain damage was induced in mice by photothrombotic cortical ischemia. The infarct volume, brain edema, neurological deficits, and motor function 24 h after ischemic injury were analyzed. We investigated BBB breakdown by measuring Evans blue extravasation in addition to endothelial cells, tight junction proteins, protease-activated receptor-1 (PAR-1), and matrix metalloproteinase-9 (MMP-9) using immunofluorescence staining and confocal microscopy. Pretreatment with Weisheng-tang significantly reduced infarct volume and edema and improved neurological and motor functions; however, Tongxuewan did not. In addition, Weisheng-tang decreased brain infarction and edema and recovered neurological and motor deficit in a dose-dependent manner (30, 100, and 300 mg/kg). Weisheng-tang pretreatment resulted in significantly less BBB damage and higher brain microvasculature after focal cerebral ischemia. Tight junction proteins, such as zonula occludens-1 (ZO-1) and claudin-5, were preserved in Weisheng-tang-pretreated mice. Moreover, the ischemic brain in these mice showed suppressed PAR-1 and MMP-9 expression. In conclusion, our findings show that Weisheng-tang, which was selected through literature analysis but has not previously been used as a stroke remedy, exerts protective effects against ischemic brain damage and suggest its possible application for potential stroke patients, especially in the elderly.

## 1. Introduction

Stroke is one of the leading causes of serious and long-term disability worldwide, although stroke mortality has been declining [1]. Treatment of stroke has been traditionally focused on reducing ischemic cell death; however, clinical trials have shown that none of the tested neuroprotective drugs achieve clinical benefit for the treatment of acute stroke [2]. The failure of clinical trials provide evidence that new therapeutic strategies for acute stroke need to be explored; one such strategy might involve the preservation of the integrity of the blood-brain barrier (BBB) [2]. The BBB is a highly selective barrier that separates the central nervous system from the peripheral circulation blood; it is formed by endothelial cells, tight junctions, astrocyte end-feet ensheathing the capillary, and pericytes embedded within the capillary basement membrane [3]. Ischemic stroke is characterized by the breakdown of the BBB and the accumulation of inflammatory infiltrates in the brain parenchyma, which can contribute to further progression of brain damage [4, 5]. Therefore, BBB disruption is recognized as a hallmark of stroke, and thus, the BBB could be a therapeutic target to reduce ischemic brain damage in patients with stroke.

Traditional medicines are typically composed of natural products, which represent a promising source of new ingredients for the development of conventional medicines. Indeed, researchers have begun to consult traditional medicine literature in the search for novel therapeutic strategies [6]. In our previous study, we investigated the effectiveness of 29 medical herbs in the treatment of stroke symptoms based on *Dongeuibogam* text-mining analysis and a mouse model of cerebral ischemia and selected the formula “Shuanghe-tang” as the candidate herbal medicinal formulas for ischemic stroke [7]. Pretreatment with Shuanghe-tang significantly reduced infarct volume and edema and improved neurological and motor functions [7]. Subsequently, we searched the traditional Korean medical literature once again to select new herbal medicinal formulas for the treatment of ischemic stroke using the revised criteria obtained from our previous research. We used the following modified criteria: (1) the formula includes at least four of the compositional herbs of Shuanghe-tang (*Paeonia lactiflora*, *Astragalus membranaceus*, *Rehmannia glutinosa*, *Angelica gigas*, *Cnidium officinale*, *Cinnamomum cassia*, and *Glycyrrhiza glabra*) and (2) the volume of these four herbs comprises over 70% of the total volume of the combined formula. Based on these criteria, we selected Tongxuewan and Weisheng-tang as novel therapeutic formulas for ischemic stroke (Table 1). According to the *Dongeuibogam*, Tongxuewan has long been used to treat the vascular diseases and Weisheng-tang has been used in individuals who suffer from exhaustion and indigestion [8].

In this study, we evaluated the protective effect of Tongxuewan or Weisheng-tang, which were selected by *Dongeuibogam* text-mining analysis, on brain damage in stroke. We hypothesized that the inhibition of BBB disruption by Tongxuewan or Weisheng-tang could be effective in ameliorating ischemic brain damage. To investigate this hypothesis, we examined the protective effect of Tongxuewan or Weisheng-tang on the parameters of ischemic brain damage and BBB disruption, such as the infarct volume, brain edema, neurological deficits, motor function, and Evans blue extravasation using the focal cerebral ischemia mouse model. In addition, we determined the expression of tight junction proteins, protease-activated receptor-1 (PAR-1), and matrix metalloproteinase-9 (MMP-9), to elucidate the mechanisms by which Weisheng-tang regulated BBB disruption.

## 2. Materials and Methods

### 2.1. Preparation of Herb Extracts

The components of Tongxuewan and Weisheng-tang (Table 1) were purchased from Hwalim Nature Drug (Busan, Korea). Tongxuewan (88 g) or Weisheng-tang (61.87 g) was boiled in 1.2 L of distilled water for 3 h; subsequently, the samples were filtered twice and concentrated using an evaporator equipped with a decompression device (EYELA Co., Tokyo, Japan). After freeze drying (Labconco, Kansas City, MO), the extracts of Tongxuewan and Weisheng-tang obtained were 21.45 g and 13.8 g, respectively.

### 2.2. Animal Experiments

Male C57BL/6 mice were purchased from Hana Biotech (Ansan, Korea). Mice were housed under a 12 h light/dark cycle and allowed *ad libitum* access to food and water. The animal protocol used in this study was reviewed by the Pusan National University-Institutional Animal Care and Use Committee (PNU-IACUC) as per their ethical procedures and scientific care, and it has been proven (PNU-2017-1477, PNU-2018-1828). Euthanasia is considered when eating less 40% of food and water, unstable breathing, and continuous for humane endpoint. We assessed and monitored the condition of the feeding and postsurgery daily for any signs of decreased activity and a decrease in food intake. We did not observe any signs of these conditions. Mice were orally administered 0.15 mL of each Tongxuewan or Weisheng-tang extract in 1x PBS at the appropriate concentration once per day for 4 days, as well as 1 h prior to focal cerebral ischemia (a total of five treatments). Experimental drugs, including PAR-1 agonist (TFLLR-NH_2_: 3 *μ*mol/kg in 40 *μ*L saline, Tocris, Bristol, UK) or control peptide (RLLFT-NH_2_: 3 *μ*mol/kg in 40 *μ*L saline, Tocris) [9], were injected into the tail vein, 30 min prior to ischemic brain injury.

### 2.3. Focal Cerebral Ischemia

Focal cerebral ischemia was induced by photothrombosis of the cortical microvessels, as previously described [7]. Briefly, mice were anesthetized with 2% isoflurane in 20% O_2_ and 80% N_2_O; subsequently, they received an intraperitoneal (i.p.) injection of rose bengal (Sigma-Aldrich, St. Louis, MO; 0.1 mL of 10 mg/mL in 0.9% saline) 5 min prior to illumination. Each mouse was fixed on a stereotaxic frame (David Kopf Instruments, Tujunga, CA), and its skull was exposed. A fiber-optic bundle containing a CL 6000 LED cold light source (Carl Zeiss, Jena, Germany) was positioned onto the sensorimotor cortex of exposed skull (2.4 mm lateral from the bregma) and illuminated for 15 min. The scalp was sutured after illumination. Subsequently, the mice were allowed to recover under a heating lamp and were returned to their home cages. Body temperature was maintained at 37.5°C during surgery using a heating pad (Harvard Apparatus, Holliston, MA).

### 2.4. Infarct Volume and Edema

The brains were removed 24 h after ischemic injury. Subsequently, infarct size was determined via 2,3,5-triphenyltetrazolium chloride (TTC) staining of 2 mm thick brain sections. Infarct size was quantified using i-Solution software (Image & Microscope Technology, Vancouver, Canada). Measurements of the direct infarct volume included areas of the ipsilateral side that had sustained direct damage. The indirect infarct volume was calculated according to the following formula: contralateral hemisphere (mm^3^)—undamaged ipsilateral hemisphere (mm^3^). Edema was calculated by subtracting the direct infarct volume from the indirect infarct volume.

### 2.5. Neurological Score

Neurological deficits were evaluated 24 h after ischemic injury using the following scoring system: 1 = turning in the direction of the ipsilateral (nondamaged) side when held by the tail, 2 = turning in the direction of the contralateral (damaged) side and difficulty bearing weight, 3 = unable to bear weight on the contralateral side, and 4 = no spontaneous movement [7].

### 2.6. Wire-Grip Test

Vestibular-motor function was evaluated 24 h after the ischemic injury using the wire-grip test. Each mouse was suspended on a metal wire and forced to hang using both forepaws. Wire grip was scored as follows: 1 = not holding onto the wire; 2 = holding onto the wire using both forepaws and hind paws but not the tail; 3 = holding onto the wire using both forepaws and hind paws as well as the tail, without movement; 4 = moving on the wire using both forepaws, both hind paws, and tail; and 5 = moving well on the wire [7].

### 2.7. Determination of Evans Blue Leakage

BBB permeability was determined by the extravasation of Evans blue. Evans blue (2% in saline, 4 mL/kg; Sigma-Aldrich) was injected into the tail vein immediately after induction of focal cerebral ischemia. One hour after injection, mice were euthanized and transcardially perfused with iced PBS. The brains were removed; subsequently, the cortical area of each hemisphere was separated, weighed, and homogenized in 400 *μ*L of N,N-dimethylformamide (Sigma-Aldrich, St. Louis, MO, USA). Following overnight incubation at 55°C, samples were centrifuged at 13,000 rpm for 30 min in 4°C. The absorbance of supernatant was measured at 620 nm via spectrophotometry, whereas Evans blue extravasation (*μ*g/g of brain tissue) was quantified using a standard curve [7].

### 2.8. Immunofluorescence Staining

Mice were perfused with cold PBS followed by 4% paraformaldehyde 24 h after focal cerebral ischemia. Immediately thereafter, brains were harvested and further fixed for 24 h in 4% paraformaldehyde; subsequently, they were cryoprotected in 30% sucrose for 72 h at 4°C. Each brain was frozen in optical cutting temperature (OCT) compound (Sakura Finetek, Torrance, CA) and stored at -80°C until analysis. The frozen brains were sectioned (thickness: 20 *μ*m) using a CM 3050 cryostat (Leica Microsystems, Wetzlar, Germany). The brain sections were immunostained with anti-CD-31 (1 : 100, BD Biosciences, Franklin Lakes, NJ), anti-ZO-1 (1 : 100, Invitrogen Corporation, Carlsbad, CA), anti-claudin-5 (1 : 100, Invitrogen Corporation), anti-PAR-1 (1 : 100, Novus, Centennial, CO), and anti-MMP-9 (1 : 100, R&D systems, Minneapolis, MN) overnight at 4°C. They were subsequently incubated with Alexa 488 or Alexa 594-conjugated secondary antibodies (1 : 500, Life Technologies, Carlsbad, CA) for 2 h in total darkness. 4′,6-Diamidino-2-phenylindole (DAPI, Molecular Probes) was used for the staining of the nuclei. Fluorescence images were visualized using a Zeiss LSM 700 laser scanning confocal device (Carl Zeiss). The images were quantified using MetaMorph Microscopy Automation and Image Analysis Software (Molecular Devices, San Jose, CA), i-Solution software (Image & Microscope Technology), and ImageJ (NIH).

### 2.9. Statistical Analysis

Data are presented as the mean ± standard error of the mean (SEM). Statistical comparisons between different groups were performed using Student's *t*-tests. Values of *p* < 0.05 were considered statistically significant.

## 3. Results

### 3.1. Pretreatment Effects of Tongxuewan or Weisheng-Tang on Ischemic Brain Damage

**Figure 1:**
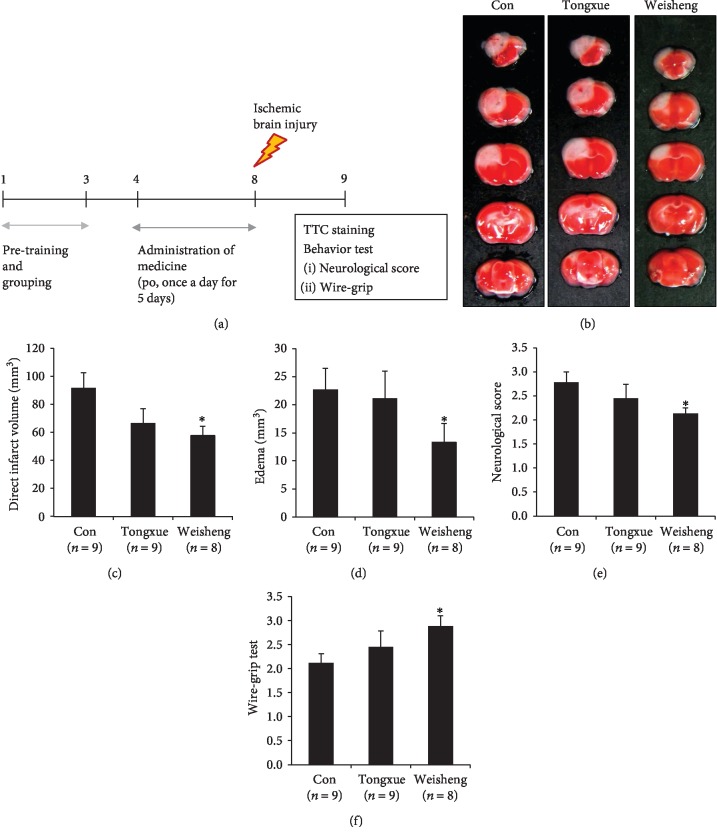
Effects of Tongxuewan and Weisheng-tang on brain infarction, edema, and behavior following ischemic brain injury. (a) Mice were pretreated via oral administration of 1000 mg/kg of Tongxuewan (*n* = 9), 500 mg/kg Weisheng-tang (*n* = 8), or PBS (control group, *n* = 9) once per day for 4 days prior to ischemic injury as well as 1 h prior to the procedure. Twenty-four hours after ischemic brain injury, the mouse brains were harvested and stained with 2% TTC solution. (b) Representative images of 2,3,5-triphenyltetrazolium chloride-stained brain coronal sections of mice. White region indicates the infarct area. (c, d) Quantification analysis of direct infarct volume (c) and edema (d). ^∗^*p* < 0.05 vs. control group. (e, f) Neurological score (e) and wire-grip tests (f) were performed to evaluate functional outcomes. ^∗^*p* < 0.05 vs. control group. TTC: 2,3,5-triphenyltetrazolium chloride.

We evaluated the pretreatment effects of Tongxuewan or Weisheng-tang on ischemic brain injury. Mice underwent oral administration of Tongxuewan (1000 mg/kg) or Weisheng-tang (500 mg/kg) once daily for 4 days prior to the induction of ischemia as well as 1 h prior to the procedure (Figure 1(a)). The outcome of the acute stroke was assessed on day 1 after focal cerebral ischemia. Direct infarct volume and edema were significantly reduced in mice treated with Weisheng-tang at a dose of 500 mg/kg, whereas Tongxuewan had no significant effect compared with control mice (Figures 1(b)–1(d)). In addition, mice treated with Weisheng-tang showed significant improvement in neurologic (Figure 1(e)) and motor (Figure 1(f)) functions compared to the control mice, suggesting that the smaller infarct volumes translated into better functional outcomes in the Weisheng-tang-treated group.

### 3.2. Dose-Dependent Effects of Weisheng-Tang on Ischemic Brain Damage

**Figure 2:**
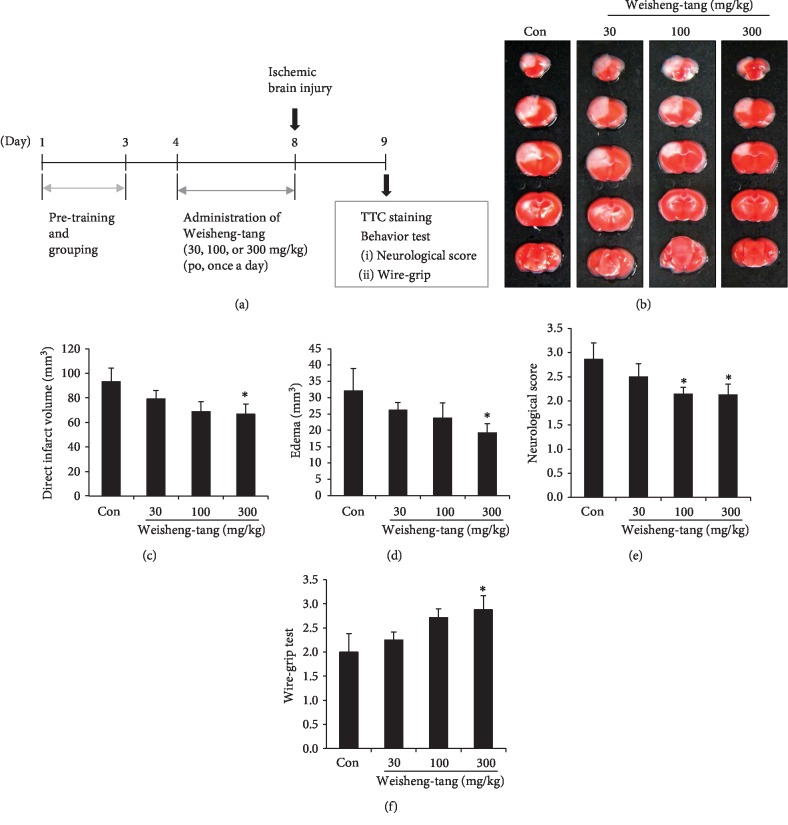
Dose-dependent effects of Weisheng-tang on ischemic brain damage. (a) Mice were pretreated via oral administration of 30, 100, or 300 mg/kg of Weisheng-tang (*n* = 7‐8 each) or PBS (control group, *n* = 7) once daily for 4 days prior to ischemic insult as well as 1 h prior to the procedure. (b) Representative photographs of coronal brain sections stained with TTC. (c, d) Quantification of direct infarct volume (c) and edema (d) 24 h postischemia. ^∗^*p* < 0.05 vs. control group. (e–f) Neurological score (e) and wire-grip (f) results were evaluated to assess recovery of neurologic deficit and vestibular motor function after ischemic brain injury. ^∗^*p* < 0.05 vs. control group. TTC: 2,3,5-triphenyltetrazolium chloride.

To determine whether Weisheng-tang exerts dose-dependent effects on ischemic brain injury, mice were pretreated with 30, 100, or 300 mg/kg of Weisheng-tang (Figure 2(a)). Our findings indicated that Weisheng-tang produced dose-dependent reductions in direct infarct and edema volume; moreover, the most significant reductions in infarct volume and edema were observed in mice pretreated with 300 mg/kg of Weisheng-tang (Figures 2(b)–2(d)). Next, we further observed dose-dependent effects of Weisheng-tang pretreatment on neurologic deficits and motor function. Significant improvements in functional outcomes were observed in mice pretreated with 300 mg/kg of Weisheng-tang, relative to those observed in the control group (Figures 2(e) and 2(f)).

### 3.3. Effects of Weisheng-Tang on BBB Disruption following Ischemic Brain Injury

**Figure 3:**
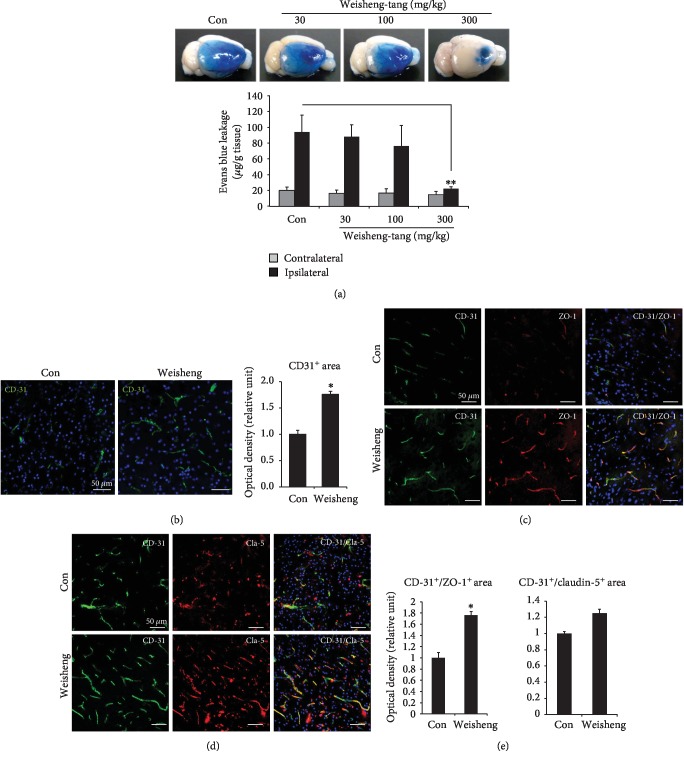
Effects of Weisheng-tang on BBB disruption following ischemic brain injury. Mice were pretreated via oral administration of 30, 100, or 300 mg/kg of Weisheng-tang (*n* = 4~6) or PBS (control group, *n* = 6) once per day for 4 days prior to ischemic insult, as well as 1 h prior to the procedure. Evans blue (4 mg/kg) was intravenously injected immediately following ischemic insult. (a) Representative photographs of Evans blue leakage in control or Weisheng-tang groups 1 h after ischemic injury and quantification of Evans blue extravasation. ^∗∗^*p* < 0.01 vs. control group. (b) Endothelial cell staining (CD31, green) shows significantly higher vessel density in the ipsilateral cerebral cortex of the Weisheng-tang-treated group (*n* = 9 in each group). ^∗^*p* < 0.05 vs. control group. Scale bar = 50 *μ*m. (c–e) Confocal images show costaining of tight junction proteins markers ZO-1 (zonula occludens-1) and claudin-5 (red) with CD31 (green) in the peri-infarct region of control and Weisheng-tang-treated mice. Mice pretreated with Weisheng-tang (300 mg/kg) exhibited increased expression of the tight junction proteins ZO-1 (c) and claudin-5 (d) following focal cerebral ischemia. Representative photographs of ZO-1 (c) and claudin-5 (d). CD31 staining for blood vessels indicated in green. Scale bar = 50 *μ*m. Quantification graphs of ZO-1 and claudin-5 immunofluorescence (e) (*n* = 9 each, ^∗^*p* < 0.05 vs. control group).

Because Weisheng-tang reduced ischemic brain edema, we evaluated the effect of Weisheng-tang on the BBB breakdown. The BBB permeability was assessed by measuring extravasation of Evans blue. Mice were pretreated with 30, 100, or 300 mg/kg of Weisheng-tang, BBB disruption was induced by photothrombotic ischemia, and Evans blue solution injected into mice extravasates through the damaged BBB (Figure 3(a)). Weisheng-tang produced dose-dependent reductions in BBB disruption, and 300 mg/kg Weisheng-tang significantly reduced BBB disruption compared to that observed in the control group (Figure 3(a)). To examine whether Weisheng-tang also affects the blood vessels in the cerebral cortex after focal cerebral ischemia, we measured the levels of blood vessel with the specific marker CD31 at the peri-infarct region (Figure 3(b)). The numbers of CD31^+^ cells were significantly greater in the Weisheng-tang-treated group than in the control group, indicating that Weisheng-tang can preserve blood vessel in the ischemic area. Next, we observed the effect of Weisheng-tang on the tight junction proteins for BBB maintenance (Figures 3(c)–3(e)). Immunofluorescence staining revealed increased levels of ZO-1 and claudin-5 following cerebral ischemia in the Weisheng-tang-treated group, suggesting that Weisheng-tang reduces the degree of BBB disruption by increasing the levels of ZO-1 and claudin-5.

### 3.4. Effects of Weisheng-Tang on PAR-1 Activation following Ischemic Brain Injury

**Figure 4:**
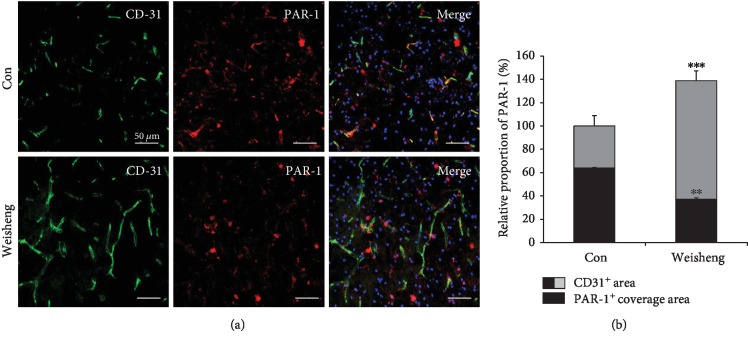
Effects of Weisheng-tang on PAR-1 levels following ischemic brain injury. (a) Confocal images show PAR-1 (red) expression coverage around the blood vessels (CD31, green) in the ischemic area. PAR-1 immunofluorescence levels were significantly reduced in mice pretreated with Weisheng-tang, although there was an increase in the CD31^+^ area. Scale bar = 50 *μ*m. (b) Quantification of CD-31^+^ area and PAR-1^+^ coverage area around the blood vessels (*n* = 6). ^∗∗^*p* < 0.01 and ^∗∗∗^*p* < 0.001 vs. control group.

**Figure 5:**
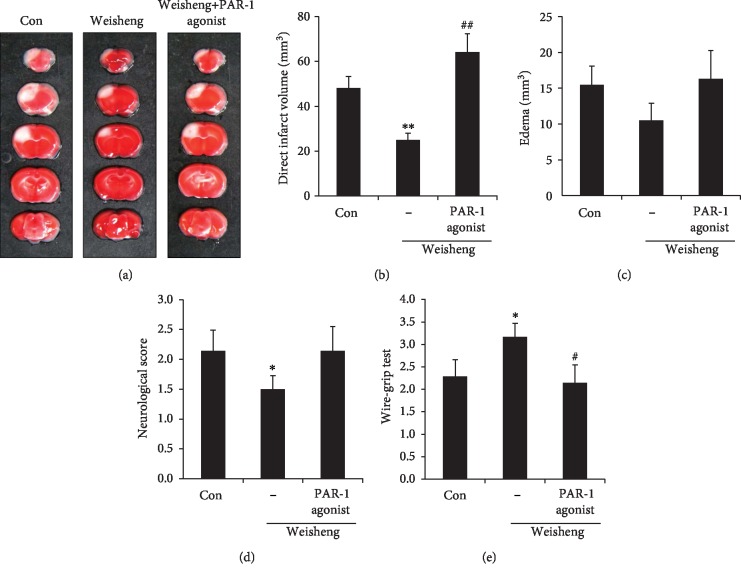
The protective effect of Weisheng-tang on ischemic brain damage was mediated by PAR-1 reduction. Mice were pretreated via oral administration of 300 mg/kg of Weisheng-tang (*n* = 7, each group) or PBS (sham group, *n* = 7) once daily for 4 days prior to ischemic insult, as well as 1 h prior to the procedure. PAR-1 agonist (TFLLR-NH_2_: 3 *μ*mol/kg in 40 *μ*L saline) or control peptide (RLLFT-NH_2_: 3 *μ*mol/kg in 40 *μ*L saline) was injected into the tail vein 30 min prior to ischemic brain injury. Twenty-four hours after focal cerebral ischemia, the mouse brains were harvested and stained with 2% TTC solution. (a) Representative photographs of brain sections stained with TTC. White region indicates the infarct area. (b–e) Quantification graphs of direct infarct volume (b) and edema (c). Neurological score (d) and wire-grip tests (e) were performed to evaluate functional outcomes. ^∗^*p* < 0.05 and ^∗∗^*p* < 0.01 vs. control group; ^#^*p* < 0.05 and ^##^*p* < 0.01 vs. control peptide cotreated group with Weisheng-tang. TTC: 2,3,5-triphenyltetrazolium chloride.

Next, we examined the effects of Weisheng-tang treatment on the expression of a specific thrombin receptor, PAR-1, which has been known to trigger the development of neuronal damage associated with ischemia and BBB breakdown [10]. In line with the results of previous studies, our results showed a high level of PAR-1 expression in the peri-infarct region after focal cerebral ischemia (data not shown), indicating that PAR-1 induction contributes to the expansion of brain damage. Despite an increase in the CD31^+^ area, Weisheng-tang pretreatment caused significant decreases in PAR-1 expression in the perivascular region (Figures 4(a) and 4(b)), suggesting that Weisheng-tang may protect brain damage via PAR-1 reduction. To confirm this, we further investigated whether PAR-1 mediated the infarct volume and edema reduction, which was functionally improved by Weisheng-tang. As seen in Figure 5, the PAR-1 agonist TFLLR-NH_2_ [9] combined with Weisheng-tang reversed the protective effect of Weisheng-tang on the infarct and edema volume and neurological and motor function (Figure 5). These findings indicate that pretreatment with Weisheng-tang decreased ischemic brain damage, possibly by PAR-1 suppression.

**Figure 6:**
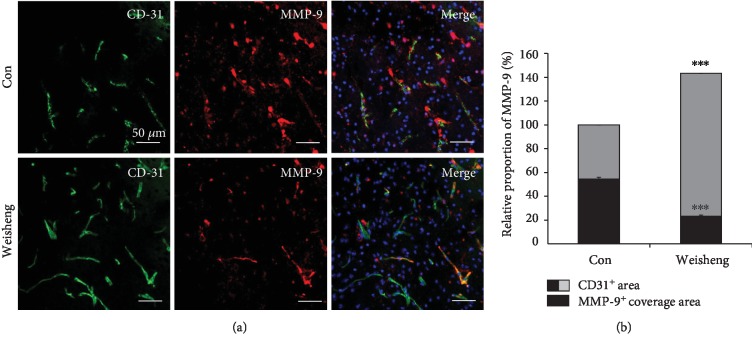
Effects of Weisheng-tang on MMP-9 production following ischemic brain injury. (a) Confocal images show MMP-9 (red) expression coverage around the blood vessels (CD31, green) in the ischemic area. MMP-9 immunofluorescence levels were significantly reduced in mice pretreated with Weisheng-tang, although there was an increase in the CD31^+^ area. Scale bar = 50 *μ*m. (b) Quantification of CD-31^+^ area and MMP-9^+^ coverage area around the blood vessels (*n* = 12). ^∗∗∗^*p* < 0.001 vs. control group.

Next, we examined the effects of Weisheng-tang treatment on the levels of the zinc-containing protease MMP-9. Mice pretreated with Weisheng-tang showed a significant reduction in MMP-9 expression in the perivascular region (Figures 6(a) and 6(b)). These results suggest that MMP-9 reduction by Weisheng-tang may contribute towards the amelioration of the BBB disruption and expansion of brain edema following ischemia.

## 4. Discussion

We evaluated the protective effects of Tongxuewan and Weisheng-tang selected by text-mining analysis of *Dongeuibogam*, the ancient Korean medical literature, on ischemic brain damage. Weisheng-tang significantly reduced ischemic brain damage; however, Tongxuewan did not. In addition, Weisheng-tang showed less BBB damage via downregulation of tight junction proteins and suppression of PAR-1 and MMP-9 in the ischemic brain. The present study suggests that the protective effect of Weisheng-tang on brain ischemic injury involves its ability to attenuate BBB disruption and expansion of brain edema.

According to the *Dongeuibogam*, Tongxuewan has long been used to treat the vascular diseases that are caused by blocked blood flow, whereas Weisheng-tang has been used in individuals who suffer from exhaustion and indigestion causing diarrhea [8]. In the present study, our findings indicated that Weisheng-tang treatment significantly reduced direct infarct and edema volume, in addition to dose-dependent increases in neurological and vestibular motor functions (Figures 1 and 2) after focal cerebral ischemia. In contrast, treatment with Tongxuewan produced no significant reductions in the extent or functional impact of ischemic injury (Figure 1). Such findings indicate that Weisheng-tang, which was screened according to certain criteria through literature analysis, exerts protective effects against ischemic brain damage, although it has not previously been used as a stroke remedy.

The blood-brain barrier (BBB) is a specialized barrier consisting of endothelial cells, tight junctions, pericytes, astrocytic end-feet processes, and the basement membrane; it is crucial in the regulation of the passage of ions, proteins, and inflammatory cells between the plasma and brain [3]. Disruption of the BBB in ischemic stroke causes dramatic changes in the chemical and cellular composition of this environment, which can contribute to further progression of brain damage [4, 5]; thus, this makes the BBB an important target to reduce brain damage in stroke. Tight junctions in the brain endothelial cells maintain BBB integrity and consist of different proteins, such as claudins and occludins [11]; in acute stroke, there is degradation of tight junctions resulting in the loss of vascular integrity [12]. We found that Weisheng-tang pretreatment significantly reduces BBB leakage and increases endothelial cells (Figures 3(a) and 3(b)). When we examined the expression pattern of ZO-1 and claudin-5 in the capillaries of the peri-infarct region, their expression pattern was clearly disrupted in the control group (Figures 3(c) and 3(d)), whereas relatively homogenous distributions and increased expression were observed in Weisheng-tang-treated mice (Figures 3(c)–3(e)). The results of the present study indicate that Weisheng-tang pretreatment reduces BBB disruption and increases levels of ZO-1 and claudin-5 in the ischemic brain.

Protease-activated receptors (PARs) are G protein-coupled receptors that convert an extracellular proteolytic cleavage event by thrombin into transmembrane signaling [13, 14]. Four members of PARs have been cloned (PAR-1, PAR-2, PAR-3, and PAR-4) in diverse neural cells of the brain [15]; however, PAR-1 is the major thrombin-activated receptor in humans [16]. Many previous studies used an animal model cerebral ischemia to determine whether pharmacological manipulation of PAR-1 signaling could provide an attractive drug discovery target for possible treatments of brain damage associated with ischemic damage and BBB breakdown [10]. PAR-1-mediated neurovascular damage during cerebral ischemia was demonstrated using *in vivo* and *in vitro* knockdown of PAR-1 [17] and an animal model of PAR-1 deficiency [18]. In addition, suppression of PAR-1 activity plays a role in the maintenance of microvascular integrity in rats undergoing subarachnoid hemorrhage [19]. Injury-induced BBB breakdown sufficient to allow extravasation of PAR-1 activators, such as thrombin, may be a result of the PAR-1-mediated mechanism underlying the pathogenesis of brain injury [20]. In the present study, we found that Weisheng-tang pretreatment significantly decreases PAR-1 expression in the perivascular region (Figures 4(a) and 4(b)); moreover, a PAR-1 agonist TFLLR-NH_2_ [9] reversed the protective effect of Weisheng-tang on ischemic brain damage (Figure 5). Collectively, these findings indicate that Weisheng-tang pretreatment may attenuate BBB dysfunction and edema following ischemic brain injury by promoting the increased expression of ZO-1 and claudin-5 and inhibition of PAR-1. However, further studies are required to investigate the mechanisms underlying the effects of Weisheng-tang on the expression of tight junction proteins and PAR-1.

Matrix metalloproteinase-9 (MMP-9) plays a key role in protease-mediated physiological and pathological changes in BBB breakdown [21, 22]. In ischemic stroke, increased MMP-9 in the damaged brain is one of the significant causes of BBB breakdown [22]; moreover, PAR-1 is a main receptor for thrombin-induced expression of MMP-9 and pathological changes in BBB breakdown [21]. BBB-constituting cells, including the brain microvascular endothelial cells, astrocytes, and brain pericytes, can release MMP-9 upon thrombin stimulation [21, 23, 24]. Lastly, we reported that treatment of PAR-1 antagonists inhibited breakdown of BBB via the downregulation of MMP-9 expression and preservation of the expression of tight junction proteins in the brain [25]. In this study, we showed Weisheng-tang pretreatment significantly increases MMP-9 expression in the perivascular region (Figures 6(a) and 6(b)), suggesting that Weisheng-tang can prevent ischemic brain injury by stabilizing the disrupted BBB via downregulation of PAR-1 and MMP-9 and upregulation of tight junction proteins.

## 5. Conclusion

In the present study, we selected two Korean herbal medicine formulas, Weisheng-tang and Tongxuewan, through *Dongeuibogam* text-mining analysis, and evaluated their protective effects on BBB disruption and brain damage in stroke. Weisheng-tang exerts protective effects against ischemic brain damage and promotes the recovery of neurological and motor function after focal cerebral ischemia, although it has not previously been used as a stroke remedy. Further experimental and clinical investigations of Weisheng-tang may aid in the development of novel therapeutic strategies for ischemic stroke.

## Figures and Tables

**Table 1:** Composition of Tongxuewan and Weisheng-tang.

Family	Scientific name	Amount (g)
Tongxuewan		
*Cnidium officinale* Makino	Cnidii Rhizoma	40
*Angelica gigas* Nakai	Angelicae Gigantis Radix	40
*Saposhnikovia divaricata* Schiskin	Saposhnikovia Radix	40
*Schizonepeta tenuifolia* Briquet	Schizonepetae Spica	40
*Rehmannia glutinosa* Libosch. var. *purpurea* Mak	Rehmanniae Radix	20
*Paeonia lactiflora* Pall.	Paeoniae Radix Rubra	20
*Glycyrrhiza glabra* Linn.	Glycyrrhizae Radix	20
	Total	220

Weisheng-tang		
*Astragalus mongholicus* Bunge	Astragali Radix	8
*Angelica gigas* Nakai	Angelicae Gigantis Radix	8
*Paeonia lactiflora* Pall.	Paeoniae Radix Alba	8
*Glycyrrhiza glabra* Linn.	Glycyrrhizae Radix	4
	Total	28

## Data Availability

The data used to support the findings of this study are available from the corresponding authors upon request.
